# Changes in public perception of artificial intelligence in healthcare after exposure to ChatGPT

**DOI:** 10.1038/s41746-025-02169-x

**Published:** 2025-11-25

**Authors:** Anders Aasted Isaksen, Jonas Rosborg Schaarup, Lasse Bjerg, Adam Hulman

**Affiliations:** 1 Steno Diabetes Center Aarhus, Aarhus University Hospital, Aarhus, Denmark; 2 Department of Public Health, Aarhus University, Aarhus, Denmark

**Keywords:** Epidemiology, Information technology

## Abstract

The public perception of artificial intelligence (AI) in healthcare is key to its large-scale acceptance and implementation. This study investigated how exposure to ChatGPT changed public perception of AI in healthcare, using baseline and follow-up data from 5899 survey participants reporting their perception of AI in 2022 (before ChatGPT’s launch) and 2024, and ChatGPT use in 2024. Multinomial multivariate logistic regression was used to model how exposure to ChatGPT use affected changes in perception of AI. At follow-up, 1195 individuals (20%) had been exposed to ChatGPT use, which was associated with higher odds of changing perception of AI to beneficial (OR 3.21 [95% CI: 2.34–4.40]) among individuals who were unsure at baseline, and lower odds of changing to uncertainty from more defined baseline perceptions. This study demonstrates the potential for reducing uncertainty and improving public perception of AI in healthcare through exposure to AI tools.

## Introduction

The implementation of artificial intelligence (AI) technology in healthcare is expected to increase efficiency, improve care quality, relieve the growing burden on healthcare systems^1^, and empower patients’ access to knowledge^2^. The practical implementation of this technology is growing at a rapid pace, as evidenced by the number of annual Food and Drug Administration approvals for AI-enabled medical devices, which increased from around five to a thousand in the last decade^3,4^. With the prospect of AI being widely implemented in healthcare settings, exposing patients and the general public to these tools, it is important to understand how first-hand experiences with AI tools influence perceptions of benefits and risks of applying AI in healthcare—particularly since a large part of the public is uncertain or undecided about their perception of AI^5–7^.

Previously, one of the challenges for the public to form a clear opinion on the application of AI was that these technologies were invisible elements of daily life^8^. In recent years, AI has become a visible, accessible, and practical tool that the public increasingly uses and interacts with in everyday life—particularly since the launch of ChatGPT in November 2022 and its rapid adoption, sometimes referred to as the “large language model revolution”^9^. With this change, the public’s perception of AI is likely to be shaped by practical experiences from first-hand use rather than intangible or theoretical considerations. Patients were quick to adopt ChatGPT and other general-purpose generative AI tools for guidance on personal healthcare conditions, highlighting the close relationship between AI and healthcare^10^. While the public’s overall perception of AI in healthcare has been studied in cross-sectional surveys across the world^5,6,11^, the evidence on the impact of first-hand exposure to AI and longitudinal changes in perception is sparse.

During discussions with members of the user panel of patients and relatives at Steno Diabetes Center Aarhus, a diabetes outpatient center in Denmark^12^, about the results of a previous survey of perception of AI in healthcare^5^, some members noted how impressed they were with ChatGPT and remarked that they had changed their perception after their first use. Inspired by this, we hypothesized that ChatGPT exposure would most strongly impact the large group of the public (one-third of survey respondents) who were unsure about AI’s benefits and risks. This study aims to provide evidence on the impact of being exposed to first-hand use of AI tools, such as ChatGPT, on the perception of AI in healthcare. Specifically, we investigate changes in public perception of AI in healthcare between 2022 and 2024, and assess the impact of exposure to ChatGPT on changes in perception from the baseline perception reported before ChatGPT’s release.

## Results

**Fig. 1:**
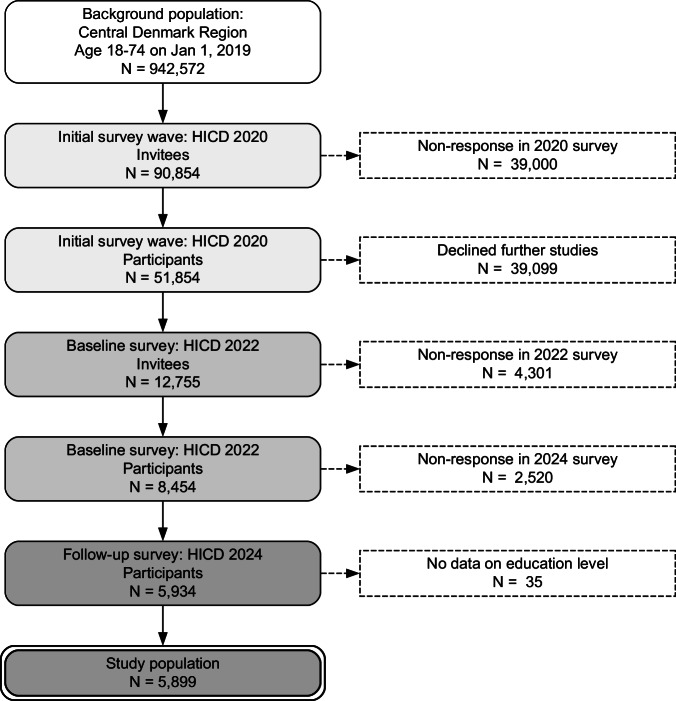
Flow of individuals from the background population of the Central Denmark Region into the two waves of the Health in Central Denmark survey and the study population. HICD Health in Central Denmark Survey.

The flow of participants from the background population into the study population is shown in Fig. 1. Of 90,854 individuals invited to the initial HICD survey, 12,755 (14%) consented to be contacted for further studies, of whom 5934 (47%) responded in both the 2022 and 2024 surveys. After excluding 35 individuals without information on education level, the study population consisted of 5899 individuals, of whom 365 (6.2%) had T1D and 2540 (43%) had T2D.

Overall characteristics of the study population and by ChatGPT exposure are shown in Table 1. At baseline, 2236 of these 5899 individuals (37%) were unsure of the benefits and risks of using AI in healthcare, while 2384 (40%) perceived the benefits as greater than the risks, 1083 (18%) perceived the benefits as equal to the risks, and 196 (3.3%) perceived the risks as outweighing the benefits.

**Table 1:** Distribution of baseline characteristics and perceptions of benefits and risks of AI in healthcare

		Overall	ChatGPT exposed	No ChatGPT exposure
Total *N* (%)		5899	1195 (20.3)	4704 (79.7)
Sex	Men	3649 (61.9)	824 (69.0)	2825 (60.1)
Age (years)	Mean (SD)	65.4 (8.9)	61.4 (10.2)	66.4 (8.2)
Diabetes status	No diabetes	2994 (50.8)	647 (54.1)	2347 (49.9)
	Type 1 diabetes	365 (6.2)	87 (7.3)	278 (5.9)
	Type 2 diabetes	2540 (43.1)	461 (38.6)	2079 (44.2)
Education level (years)	<10	289 (4.9)	17 (1.4)	272 (5.8)
	10–15	3334 (56.5)	530 (44.4)	2804 (59.6)
	>15	2276 (38.6)	648 (54.2)	1628 (34.6)
Perception of AI at baseline	Don’t know	2236 (37.9)	226 (18.9)	2010 (42.7)
	Benefits	2384 (40.4)	735 (61.5)	1649 ((35.1)
	Equal	1083 (18.4)	202 (16.9)	881 (18.7)
	Risks	196 (3.3)	32 (2.7)	164 (3.5)
Perception of AI at follow-up	Don’t know	2325 (39.4)	205 (17.2)	2120 (45.1)
	Benefits	2044 (34.6)	666 (55.7)	1378 (29.3)
	Equal	1132 (19.2)	238 (19.9)	894 (19.0)
	Risks	398 (6.7)	86 (7.2)	312 (6.6)

Unweighted distributions.

At follow-up, 1195 individuals (20%) had been exposed to ChatGPT use. These individuals tended to be younger (mean age 61 years vs. 66 years among unexposed), had a higher proportion of men (69% vs. 60%), a lower proportion with diabetes (46% vs. 50%), and had a higher education level (54% vs. 35% had completed more than 15 years of education) than those unexposed. Only 155 (13%) of the 1195 ChatGPT-exposed individuals reported using ChatGPT often, while 1223 (26%) of the 4704 unexposed individuals reported having never heard about ChatGPT.

At baseline, the group that was subsequently exposed to ChatGPT had a higher proportion of individuals who perceived benefits of AI in healthcare (62% vs. 35%) and a lower proportion of individuals unsure of their perception (19% vs. 43%) than the unexposed group. The two groups differed less in terms of proportions of individuals reporting “Equal” (17% vs. 19%) or “Risks” (2.7% vs. 3.5%). Study population characteristics weighted against the background population are available in Supplementary Table 1, and characteristics stratified by baseline perception are available in Supplementary Table 2.

**Fig. 2:**
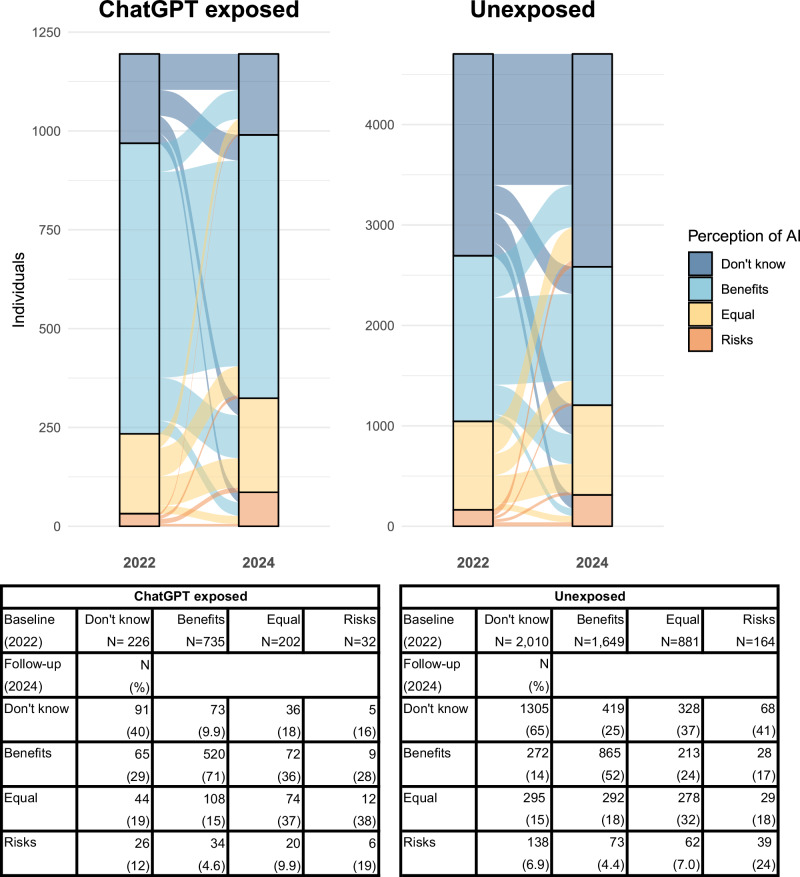
Changes in distributions of perception from baseline to follow-up by ChatGPT exposure. Changes from baseline (2022) to follow-up (2024) among individuals exposed to ChatGPT use (left) and those unexposed (right) in alluvial plots (top) and cross-tables (bottom). Note the differing y-axes. Unweighted counts.

In the follow-up survey, the overall proportion of individuals reporting “Benefits” decreased to 35%, while the proportion perceiving “Risks” doubled to 6.7%. Among individuals who reported “Don’t know” at baseline, 65 (29%) of the 226 who were subsequently exposed to ChatGPT use changed perception to “Benefits”, compared to 272 (14%) of the 2010 unexposed individuals from this baseline group (Fig. 2). Changes away from a positive perception also occurred: among those who reported “Benefits” at baseline, 520 (71%) of the 735 who were subsequently exposed to ChatGPT maintained this perception at follow-up, compared to just 865 (52%) of the 1649 unexposed.

**Fig. 3:**
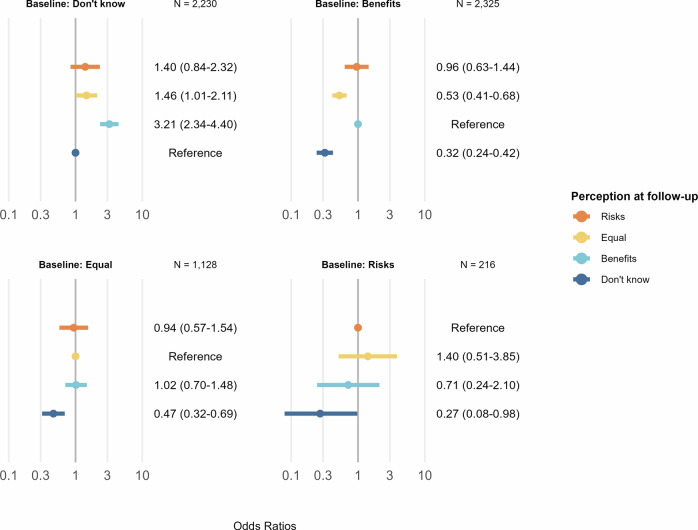
Odds ratios of changes in perception from baseline to follow-up associated with exposure to ChatGPT use stratified by baseline perception. Estimates from a multinomial logistic regression model adjusted for age, sex, education level, and diabetes status, and weighted with stabilized propensity score weights based on the distributions of these covariates in the background population. Note that the outcome reference category differs between the strata to match the baseline perception level.

Figure 3 presents the odds ratios (ORs) for changing perception versus maintaining baseline perception at follow-up associated with exposure to ChatGPT use (e.g., an OR > 1 indicates a higher likelihood of change in perception among individuals exposed to ChatGPT compared to unexposed individuals) for each baseline perception group. Exposure to ChatGPT use was associated with higher odds of changing perception to “Benefits” (OR 3.21 [95% CI: 2.34–4.40]) or to “Equal” (OR 1.46 [1.01–2.11]) vs. maintaining the baseline perception in individuals who reported “Don’t know” at baseline. In the remaining baseline perception groups, exposure to ChatGPT use was associated with lower odds of changing perception to “Don’t know” vs. maintaining the baseline perception at follow-up among individuals reporting “Benefits” (OR 0.32 [0.24–0.42]), “Equal” (OR 0.47 [0.32–0.69]) or “Risks” (OR 0.27 [0.08–0.98]) at baseline.

In supplementary analyses (Supplementary Figs. 2 and 3) using unweighted models and models weighted to the characteristics of invitees to the initial HICD survey, the estimates for ChatGPT exposure were consistent with the main analysis. However, the estimates for change of perception from “Don’t know” at baseline towards “Risks” (OR 2.35 [1.45-3.81]), “Equal” (OR 1.84 [1.25-2.73]), and “Benefit” (OR 2.92 [2.04-4.17]) at follow-up differed in magnitude between the unweighted and the main analyses.

Some baseline characteristics were associated with change of perception at follow-up in the regression models (Table 2). Generally, shorter length of education, older age, and female sex were associated with lower odds of changing baseline perception from “Don’t know” to a more defined perception at follow-up—especially to “Benefits”, and these groups were also more likely to change perception to “Don’t know” at follow-up from other baseline perceptions. In overall analyses adjusted for—rather than stratified by—baseline perception, exposure to ChatGPT use was associated with lower odds of reporting “Don’t know”, “Equal” or “Risks” than “Benefits” at follow-up (Supplementary Table 3).

**Table 2:** Full list of risk factors for change in perception of AI from baseline to follow-up

Baseline perception	Follow-up perception	Variable (value)	Estimate (95% CI)
Don’t know	Benefits	ChatGPT use (exposed)	3.21 (2.34–4.40)
Don’t know	Benefits	Sex (women)	0.43 (0.33–0.57)
Don’t know	Benefits	Age (per 10 years)	0.86 (0.75–0.98)
Don’t know	Benefits	Diabetes (Type 1)	1.30 (0.44–3.82)
Don’t know	Benefits	Diabetes (Type 2)	0.82 (0.55–1.23)
Don’t know	Benefits	Education level (10–15)	3.01 (1.68–5.39)
Don’t know	Benefits	Education level ( >15)	8.03 (4.36–14.80)
Don’t know	Equal	ChatGPT use (exposed)	1.46 (1.01–2.11)
Don’t know	Equal	Sex (women)	0.80 (0.62–1.04)
Don’t know	Equal	Age (per 10 years)	0.98 (0.85–1.12)
Don’t know	Equal	Diabetes (Type 1)	1.73 (0.63–4.77)
Don’t know	Equal	Diabetes (Type 2)	1.19 (0.84–1.67)
Don’t know	Equal	Education level (10–15)	1.34 (0.90–1.99)
Don’t know	Equal	Education level ( >15)	2.15 (1.37–3.37)
Don’t know	Risks	ChatGPT use (exposed)	1.40 (0.84–2.32)
Don’t know	Risks	Sex (women)	0.84 (0.58–1.22)
Don’t know	Risks	Age (per 10 years)	0.79 (0.66–0.95)
Don’t know	Risks	Diabetes (Type 1)	1.01 (0.20–5.04)
Don’t know	Risks	Diabetes (Type 2)	1.65 (1.07–2.54)
Don’t know	Risks	Education level (10–15)	1.67 (0.90–3.10)
Don’t know	Risks	Education level ( >15)	1.68 (0.82–3.44)
Benefits	Don’t know	ChatGPT use (exposed)	0.32 (0.24–0.42)
Benefits	Don’t know	Sex (women)	2.24 (1.77–2.84)
Benefits	Don’t know	Age (per 10 years)	1.14 (1.00–1.29)
Benefits	Don’t know	Diabetes (Type 1)	1.68 (0.69–4.12)
Benefits	Don’t know	Diabetes (Type 2)	1.32 (0.93–1.89)
Benefits	Don’t know	Education level (10–15)	1.38 (0.79–2.41)
Benefits	Don’t know	Education level ( >15)	0.80 (0.45–1.43)
Benefits	Equal	ChatGPT use (exposed)	0.53 (0.41–0.68)
Benefits	Equal	Sex (women)	1.88 (1.49–2.37)
Benefits	Equal	Age (per 10 years)	0.86 (0.77–0.97)
Benefits	Equal	Diabetes (Type 1)	1.22 (0.54–2.74)
Benefits	Equal	Diabetes (Type 2)	1.10 (0.76–1.58)
Benefits	Equal	Education level (10–15)	2.78 (1.24–6.26)
Benefits	Equal	Education level ( >15)	1.77 (0.78–4.04)
Benefits	Risks	ChatGPT use (exposed)	0.96 (0.63–1.44)
Benefits	Risks	Sex (women)	1.15 (0.75–1.77)
Benefits	Risks	Age (per 10 years)	0.71 (0.58–0.86)
Benefits	Risks	Diabetes (Type 1)	2.02 (0.72–5.67)
Benefits	Risks	Diabetes (Type 2)	1.55 (0.90–2.70)
Benefits	Risks	Education level (10–15)	1.28 (0.40–4.06)
Benefits	Risks	Education level ( >15)	0.53 (0.16–1.77)
Equal	Benefits	ChatGPT use (exposed)	1.02 (0.70–1.48)
Equal	Benefits	Sex (women)	0.82 (0.59–1.14)
Equal	Benefits	Age (per 10 years)	1.24 (1.06–1.46)
Equal	Benefits	Diabetes (Type 1)	3.20 (0.61–16.72)
Equal	Benefits	Diabetes (Type 2)	1.42 (0.85–2.37)
Equal	Benefits	Education level (10–15)	1.65 (0.83–3.31)
Equal	Benefits	Education level ( >15)	2.53 (1.21–5.28)
Equal	Don’t know	ChatGPT use (exposed)	0.47 (0.32–0.69)
Equal	Don’t know	Sex (women)	1.18 (0.87–1.59)
Equal	Don’t know	Age (per 10 years)	1.09 (0.95–1.26)
Equal	Don’t know	Diabetes (Type 1)	3.13 (0.70–14.01)
Equal	Don’t know	Diabetes (Type 2)	1.61 (1.03–2.54)
Equal	Don’t know	Education level (10–15)	0.96 (0.55–1.67)
Equal	Don’t know	Education level ( >15)	0.79 (0.43–1.47)
Equal	Risks	ChatGPT use (exposed)	0.94 (0.57–1.54)
Equal	Risks	Sex (women)	0.88 (0.56–1.37)
Equal	Risks	Age (per 10 years)	0.73 (0.61–0.88)
Equal	Risks	Diabetes (Type 1)	1.95 (0.26–14.68)
Equal	Risks	Diabetes (Type 2)	1.21 (0.61–2.43)
Equal	Risks	Education level (10–15)	0.64 (0.26–1.60)
Equal	Risks	Education level ( >15)	1.09 (0.41–2.84)
Risks	Equal	ChatGPT use (exposed)	1.40 (0.51–3.85)
Risks	Equal	Sex (women)	1.12 (0.47–2.70)
Risks	Equal	Age (per 10 years)	0.79 (0.45–1.39)
Risks	Equal	Diabetes (Type 1)	0.12 (0.00–24.43)
Risks	Equal	Diabetes (Type 2)	0.88 (0.23–3.33)
Risks	Equal	Education level (10–15)	16.33 (0.75–355.85)
Risks	Equal	Education level ( >15)	6.35 (0.27–148.94)
Risks	Benefits	ChatGPT use (exposed)	0.71 (0.24–2.10)
Risks	Benefits	Sex (women)	0.36 (0.14–0.91)
Risks	Benefits	Age (per 10 years)	0.38 (0.21–0.68)
Risks	Benefits	Diabetes (Type 1)	0.02 (0.00–15.77)
Risks	Benefits	Diabetes (Type 2)	0.21 (0.04–1.18)
Risks	Benefits	Education level (10–15)	1.68 (0.33–8.63)
Risks	Benefits	Education level ( >15)	0.96 (0.16–5.56)
Risks	Don’t know	ChatGPT use (exposed)	0.27 (0.08–0.98)
Risks	Don’t know	Sex (women)	1.55 (0.65–3.67)
Risks	Don’t know	Age (per 10 years)	0.77 (0.44–1.33)
Risks	Don’t know	Diabetes (Type 1)	0.73 (0.03–20.67)
Risks	Don’t know	Diabetes (Type 2)	0.94 (0.28–3.17)
Risks	Don’t know	Education level (10–15)	2.11 (0.53–8.48)
Risks	Don’t know	Education level ( >15)	0.30 (0.06–1.53)

Estimates of odds ratios (ORs) from multivariate multinomial regression models with 95% confidence intervals (CI) vs. the following reference levels of each categorical variable: ChatGPT: unexposed, sex: men, diabetes: no diabetes, education level: < 10 years.

## Discussion

In this study, we found that exposure to ChatGPT reduced the uncertainty of perceived benefits and risks of AI applied in healthcare, regardless of the perception of AI prior to ChatGPT exposure. Furthermore, we found that exposure to ChatGPT mainly resulted in a change towards a positive perception of AI in healthcare among individuals who were uncertain prior to this exposure. Overall, the perception of AI in healthcare became more negative between 2022 and 2024, with a substantial decrease in the proportion of individuals perceiving mainly benefits and more than a doubling of those perceiving mainly risks. These trends were present in both the ChatGPT-exposed and unexposed groups, but were less pronounced among individuals exposed to ChatGPT.

Overall, exposure to ChatGPT use was associated with shifts toward more positive perceptions of AI compared to non-exposure. This was driven by shifts from uncertainty at baseline to positive perception at follow-up, without any evidence of changes toward more positive or skeptical perceptions in individuals with other baseline perceptions. Unweighted analyses showed a more diverse influence of ChatGPT exposure in the uncertain group, with a higher likelihood of shifting towards both risks and benefits. The study population was older than the background population due to the HICD survey invites being sampled based on the age distribution of individuals with diabetes, and this difference between the weighted and unweighted analyses may be due to a different influence of ChatGPT use among older uncertain individuals than among younger.

Uptake of this technology in the public was limited, as only one-in-five survey respondents had tried ChatGPT (and one-in-five had not even heard of it). Uptake was particularly poor among people with shorter education, women, and older age groups—characteristics which were all associated with changes towards negative or uncertain perceptions of AI or maintaining these skeptical perceptions from baseline to follow-up. This finding adds to previous reports of more skeptical outlooks on AI among individuals with low digital familiarity in the United Kingdom (UK)^13^. Thus, improving digital literacy in these demographics may reduce uncertainty and skepticism regarding the benefits and risks of AI in healthcare.

As current and future patients, the public is a key stakeholder in the implementation of AI in the healthcare system, and their perception of the benefits and risks of the technology may be critical to large-scale implementation and adoption of this technology^14^. Public perception of the technology remained largely positive, with only a small minority perceiving risks as outweighing benefits at follow-up, but the growing skepticism towards AI in healthcare may become a concern if this trend grows in the coming years. Our study suggests that exposure to AI tools like ChatGPT influenced public perception of AI and could help mitigate growing skepticism, particularly as nearly half of the ChatGPT-unexposed group was undecided at follow-up.

Our study demonstrates the potential for changing public perceptions of AI in healthcare by reducing uncertainty among individuals through exposure to AI tools like ChatGPT. In most contexts, uncertainty is psychologically closer to skepticism and negative emotional states than to optimism^15,16^. Thus, “Don’t know” responses should not be interpreted as neutral, and reducing this uncertainty is valuable, even if it leads some individuals to form a negative opinion. Although an increase in explicitly negative perceptions may seem counterproductive, it is, at the magnitude observed in our study, arguably preferable to widespread disengagement and uncertainty among the public. This transition from uncertainty to a definite stance—positive or negative—is a necessary step toward a more mature and informed societal conversation about how AI, and emerging technologies more broadly, should be governed and integrated into healthcare systems. To our knowledge, this is the first study to report how exposure to an AI tool directly impacts public perceptions of AI in healthcare, as the few existing studies on changes in perception after exposure to AI were limited to specific groups, such as healthcare professionals or patients. For instance, emergency radiology professionals reported reduced uncertainty and increased perceived benefits of AI following its implementation^17^, while urolithiasis patients exposed to ChatGPT-generated notes experienced a negative shift in attitudes^18^. Thus, there is a lack of evidence to guide the implementation of AI in healthcare, which is seeing increasing uptake across areas of healthcare and data modalities, such as electronic health record analysis for clinical diagnostics and prediction^19^, clinical image classification^20,21^, and voice recordings of patients for out-of-hours cardiac arrest detection^22^ and ambient AI scribes^23^.

We observed a growing skepticism toward AI in healthcare from 2022 to 2024 in our study, similar to trends in the UK’s *Public Attitudes to Data and AI Tracker Survey*, which found that the public became more pessimistic about the impact of AI on society from 2021 to 2023, partly due to data and privacy concerns^13^. These findings contrast with a previous study based on data from social media, which found a positive trend in sentiment towards AI in medical imaging over time from 2019 to 2024^24^. This inconsistency may be due to differences in methodology and settings, but it may also reflect that the public’s perception of AI is subject to external factors such as media coverage of AI and digital technology. The sentiment of media coverage may change substantially over time, geography, and media source. Within research on the medical knowledge of ChatGPT, several reports have praised it for matching or exceeding the quality of human doctors in medical licensing exams^25–28^ and for showing higher levels of empathy in written communication with patients^29^. In contrast, other studies have highlighted how ChatGPT may perform significantly worse than human doctors in more complicated, free-text clinical case exams^30^, and how minor nuances in the wording of inputs can lead to highly biased outputs^31^. Depending on which of these messages gets picked up by the latest mainstream media coverage, the public perception may shift according to this, rather than the public’s first-hand experiences with AI.

The strengths of this study include the large study population, which allowed for stratified analyses to identify specific changes rather than an overall effect associated with exposure to ChatGPT use. As the baseline perception data was collected shortly before the launch of ChatGPT, this allowed us to closely study the changes in perception after exposure to ChatGPT. Finally, the population-wide register data enabled weighted analyses to increase the representativeness of results and reduce the risk of selection bias inherent in survey-based studies.

There are some limitations to the study. Due to the observational nature of the study, confounding cannot be ruled out. As we did not assess all potential confounders of change in perception of AI that may have been related to personal use of ChatGPT, e.g., exposure to other AI tools or awareness of positive or negative media reports on use of AI or digital technology in healthcare, we cannot rule out that such external factors biased our results. Also, while the question on perception of AI in healthcare was similar in the baseline and follow-up surveys, the preceding content of the questionnaires differed, which could bias responses due to context or fatigue effects^32^. Another limitation of this study is that the population represented the general public, who likely use ChatGPT as a general-purpose tool rather than for healthcare-specific purposes. While diabetes status did not influence the results, the findings may not apply to all patient populations or clinical AI use cases. Public perception of healthcare-specific AI tools likely depends on the tool, its user experience, and its context—factors beyond the scope of this study. Furthermore, we acknowledge that our survey question regarding “AI in healthcare” is broad and does not capture the nuances of public perception across specific applications. However, this broad framing offers a distinct advantage by capturing the public’s overall perception, the societal zeitgeist, which may be more influential in shaping public discourse and guiding high-level policy decisions than opinions on specific, technical use-cases that may be unfamiliar to much of the public. While granular studies on specific applications are important, understanding this baseline, general sentiment is a critical first step.

Our findings suggest that a logical next step toward enhancing public receptiveness to AI in healthcare is to strategically increase public exposure to high-value AI applications. Our study used ChatGPT, an AI tool that many find genuinely useful, and considering the mixed findings in the literature—where some AI exposures have shaped perceptions in a negative direction—the quality of the exposure is likely crucial. Evidence on this dynamic remains sparse (Supplementary Note 1), and more research is necessary to understand how some qualities and implementations of an AI tool enhance or decrease the public’s receptiveness to this technology. Ultimately, this represents only one potential component of a broader strategy, which should also include efforts to increase eHealth literacy and empower patients to make informed decisions about the use of AI in their care.

In conclusion, exposure to ChatGPT was associated with a change towards a positive perception of the benefits and risks of AI in healthcare among individuals who were uncertain prior to exposure. Individuals with more defined perceptions of AI prior to exposure to ChatGPT were not swayed towards positive or negative perceptions, but were less likely to become uncertain after exposure to ChatGPT. Overall, the perception of benefits declined between 2022 and 2024, but exposure to ChatGPT mitigated this trend. Public acceptance of AI in healthcare is key to large-scale implementation of the technology, and while we found increasing skepticism towards AI in healthcare among the public over time, this study demonstrates the potential for changing the public perception of AI in healthcare through exposure of the public to AI tools.

## Methods

### Data collection

In this study, we collected data on the perception of AI and ChatGPT use and combined it with administrative data from nationwide Danish registers.

Survey data: *Health in Central Denmark* (HICD) is a digital and postal questionnaire survey initially conducted in 2020 on all inhabitants of the Central Denmark Region aged 18 to 74 years identified as prevalent diabetes cases in register data on 31 December 2018, plus an equally-sized group of people without diabetes (matched to diabetes cases by sex, age, and municipality)^33^. Among respondents in 2020 who volunteered to be contacted for further studies, a baseline survey on perception of AI was conducted in the spring of 2022^34^, and these individuals were contacted again in 2024 as part of the next wave of the HICD survey, which provided follow-up data for this study^35^.

Perceived risks and benefits of AI in healthcare were assessed in baseline (2022) and follow-up (2024) surveys. Participants were asked, “Do you think the benefits of using artificial intelligence in the healthcare sector outweigh the risks?” with four answers: “Benefits outweigh risks” (“Benefits”), “Benefits equal risks” (“Equal”), “Risks outweigh benefits” (“Risks”), and “Don’t know” (the 2024 questionnaire contained two “don’t know”-responses options that were merged for this study)^35^. In the 2022 questionnaire, participants who reported having never heard of AI were not subsequently asked about their perception, but were classified as “Don’t know”. Information on exposure to ChatGPT use was reported in 2024 with the question “Have you used ChatGPT?” with the following answer options: “Yes, I use it often”, “Yes, I have tried it”, “No, but I have heard about it”, or “No, I have not heard about it”. In the analyses, this variable was included as binary: “ChatGPT exposed” and “Unexposed”, by merging the two former and latter categories, respectively.

Administrative and healthcare data covering the entire Danish population can be linked at the individual level, which enables weighting of analyses from the HICD survey to the background population^36^. Information on age, sex, and education level (defined by the total length of attained education) was obtained from the *Danish Civil Registration System*^37^ and the *Danish Population Education Register*^38^. Diabetes status was defined as prevalent “Type 1 diabetes” (T1D), “Type 2 diabetes” (T2D), or “No diabetes” at baseline according to the Open-Source Diabetes Classifier based on HbA1c measurements ≥48 mmol/mol (6.5%), hospital diagnoses of diabetes, diabetes-specific podiatrist services, and purchases of glucose-lowering drugs^39^.

This study was approved by Statistics Denmark and the Danish Health Data Authority as part of the HICD project, registered in the Central Denmark Region internal register of research projects (reg. no. 1-16-02-165-20). Informed consent was collected from participants in the HICD survey. In Denmark, observational research based exclusively on questionnaire and register data, such as this study, is exempt from further ethical approval.

### Statistical analysis

The distribution of study population characteristics was tabulated by exposure to ChatGPT use and reported as frequencies (%) or mean (SD). Alluvial plots and cross-tabulations were applied to visualize raw changes in perceptions of AI from baseline to follow-up between ChatGPT-exposed and unexposed individuals. Multinomial logistic regression was used to model the association between ChatGPT use and the perception of AI use in healthcare in the follow-up survey, stratified by perception of AI in the baseline survey. To assess the change in perception from baseline, the reference category of the outcome differed between each stratum to reflect the baseline perception in each group. The models were adjusted for age (continuous), sex, education level (“<10 years”, “10–15 years”, and “>15 years”), and diabetes status. To account for differential survey participation by demographic and clinical characteristics, we computed inverse probability weights to align the distribution of respondents with that of the background population. Specifically, we estimated each individual’s probability of survey response in a logistic regression model using age, sex, education level, and diabetes status as independent variables on all 942,572 individuals in the background population. These probabilities were used to compute stabilized weights for each respondent. Finally, the stabilized weights were truncated at the 95th percentile to reduce the influence of extreme values^40^.

Unweighted descriptive results are shown in the main text (Table 1; Figs. 1, 2) to illustrate the observed survey data, whereas weighted descriptive results—adjusted to the background population—are provided in supplementary material (Supplementary Table 1 and Supplementary Fig. 1). The main regression analyses were estimated using weighted data (Fig. 3, Table 2). In supplementary analyses, we (i) fitted unweighted models and (ii) repeated the weighting procedure using the 90,854 individuals invited to the 2020 HICD survey as the reference population instead of the full background population (Supplementary Figs. 2 and 3). Additional sensitivity analyses included unstratified models adjusted for baseline perception (Supplementary Table 3). Statistical analyses were pre-registered^41^ and performed using *R* version 4.4.1^42^ and packages for data processing^43^, weighting^44^, regression modeling^45^, and visualizations^46,47^.

## Supplementary information

Supplementary Information

## Data Availability

The Health in Central Denmark data is hosted on remote servers at Statistics Denmark. The project is managed by a steering committee at Steno Diabetes Center Aarhus, Aarhus University Hospital. The steering committee encourages interested researchers to use this resource. More information is available on the website: https://www.stenoaarhus.dk/research/resources/health-in-central-denmark/.

## Code availability

The code for this study is available on GitHub at https://github.com/hulmanlab/chatgpt_perception_ai.
