# Supplementary material for: Changes in public perception of artificial intelligence in healthcare after exposure to ChatGPT
Source: NPJ Digit Med. 2025 Nov 25;8:795. doi: 10.1038/s41746-025-02169-x (PMC12749003; doi:10.1038/s41746-025-02169-x)
Supplement: Supplementary file 1 — Supplementary Information [file 41746_2025_2169_MOESM1_ESM.pdf]

Supplementary Information:

Supplementary Table 1: Study population characteristics by ChatGPT exposure status, weighted to background population by sex, age, education level and diabetes status.

		Overall	ChatGPT exposed	No ChatGPT exposure
Total N (%)		5899	1468 (24.9)	4431 (75.1)
Sex	Men	2948 (50.0)	791 (53.9)	2157 (48.7)
Age (years)	Mean (SD)	60.7 (10.3)	55.9 (10.6)	62.3 (9.7)
Diabetes status	No diabetes	5095 (86.4)	1294 (88.1)	3801 (85.8)
	Type 1 diabetes	88 (1.5)	30 (2.1)	57 (1.3)
	Type 2 diabetes	716 (12.1)	144 (9.8)	572 (12.9)
Education level (years)	< 10	474 (8.0)	37 (2.5)	437 (9.9)
	10 - 15	3441 (58.3)	689 (46.9)	2752 (62.1)
	> 15	1984 (33.6)	742 (50.6)	1242 (28.0)
Perception of AI at baseline	Don't know	2230 (37.8)	297 (20.2)	1933 (43.6)
	Benefits	2325 (39.4)	882 (60.1)	1443 (32.6)
	Equal	1128 (19.1)	245 (16.7)	883 (19.9)
	Risks	216 (3.7)	44 (3.0)	172 (3.9)
Perception of AI at follow-up	Don't know	2260 (38.3)	260 (17.7)	2000 (45.1)
	Benefits	2023 (34.3)	795 (54.2)	1228 (27.7)
	Equal	1204 (20.4)	298 (20.3)	905 (20.4)
	Risks	412 (7.0)	114 (7.8)	298 (6.7)

Supplementary Table 2: Study population characteristics by baseline perception of AI

	Baseline perception	Don't know	Benefits	Equal	Risks
Total N (%)		2236 (37.9)	2384 (40.4)	1083 (18.4)	196 (3.3)
Sex	Men	1069 (47.8)	1782 (74.7)	676 (62.4)	122 (62.2)
Age (years)	Mean (SD)	66.4 (8.3)	64.6 (9.1)	65.2 (9.3)	64.9 (8.7)
Diabetes status	No diabetes	1073 (48.0)	1260 (52.9)	558 (51.5)	103 (52.6)
	Type 1 diabetes	131 (5.9)	163 (6.8)	59 (5.4)	12 (6.1)
	Type 2 diabetes	1032 (46.2)	961 (40.3)	466 (43.0)	81 (41.3)
Education level (years)	< 10	172 (7.7)	48 (2.0)	57 (5.3)	12 (6.1)
	10 - 15	1433 (64.1)	1162 (48.7)	622 (57.4)	117 (59.7)
	> 15	631 (28.2)	1174 (49.2)	404 (37.3)	67 (34.2)
Perception of AI at follow-up	Don't know	1396 (62.4)	492 (20.6)	364 (33.6)	73 (37.2)
	Benefits	337 (15.1)	1385 (58.1)	285 (26.3)	37 (18.9)
	Equal	339 (15.2)	400 (16.8)	352 (32.5)	41 (20.9)
	Risks	164 (7.3)	107 (4.5)	82 (7.6)	45 (23.0)
ChatGPT use	Exposed	226 (10.1)	735 (30.8)	202 (18.7)	32 (16.3)

Supplementary Figure 1: Changes in distributions of perception from baseline to follow-up by ChatGPT exposure

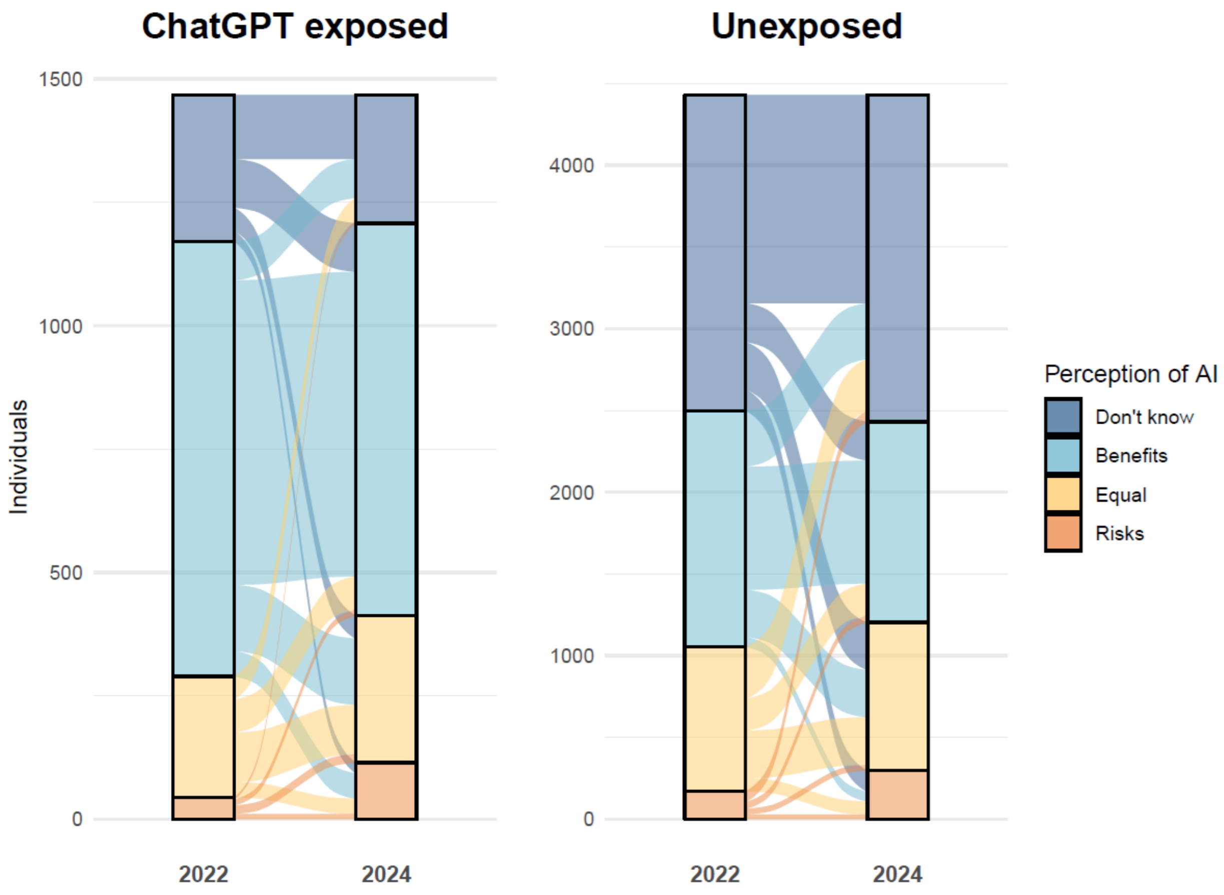

ChatGPT exposed				
Baseline (2022)	Don't know N= 298	Benefits N=882	Equal N=245	Risks N=44
Follow-up (2024)	N (%)			
Don't know	130 (44)	79 (9)	47 (19)	4 (9)
Benefits	99 (33)	617 (70)	67 (27)	12 (27)
Equal	47 (16)	134 (15)	100 (41)	17 (39)
Risks	22 (7.4)	52 (5.9)	31 (13)	11 (25)

Unexposed				
Baseline (2022)	Don't know N= 1,933	Benefits N=1,443	Equal N=884	Risks N=171
Follow-up (2024)	N (%)			
Don't know	1,274 (66)	342 (24)	318 (36)	65 (38)
Benefits	238 (12)	752 (52)	196 (22)	42 (25)
Equal	291 (15)	289 (20)	291 (33)	35 (20)
Risks	130 (6.7)	60 (4.2)	79 (8.9)	29 (17)

Changes from baseline (2022) to follow-up (2024) among individuals exposed to ChatGPT use (left) and those unexposed (right) in alluvial plots (top) and cross-tables (bottom). Note the differing Y-axes. Counts weighted to background population

Supplementary Figure 2: Odds ratios of changes in perception from baseline to follow-up associated with exposure to ChatGPT use stratified by baseline perception.

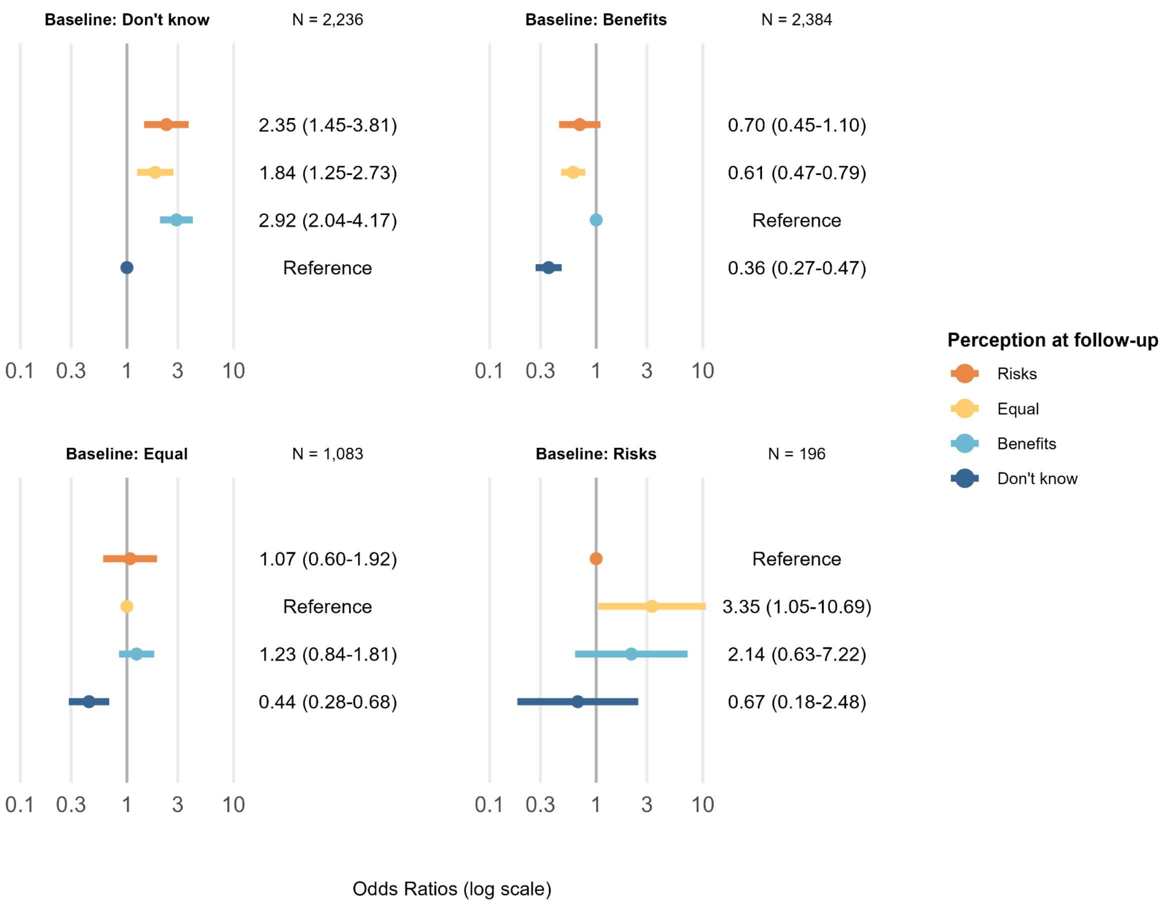

Unweighted analyses.

Supplementary Figure 3: Odds ratios of changes in perception from baseline to follow-up associated with exposure to ChatGPT use stratified by baseline perception.

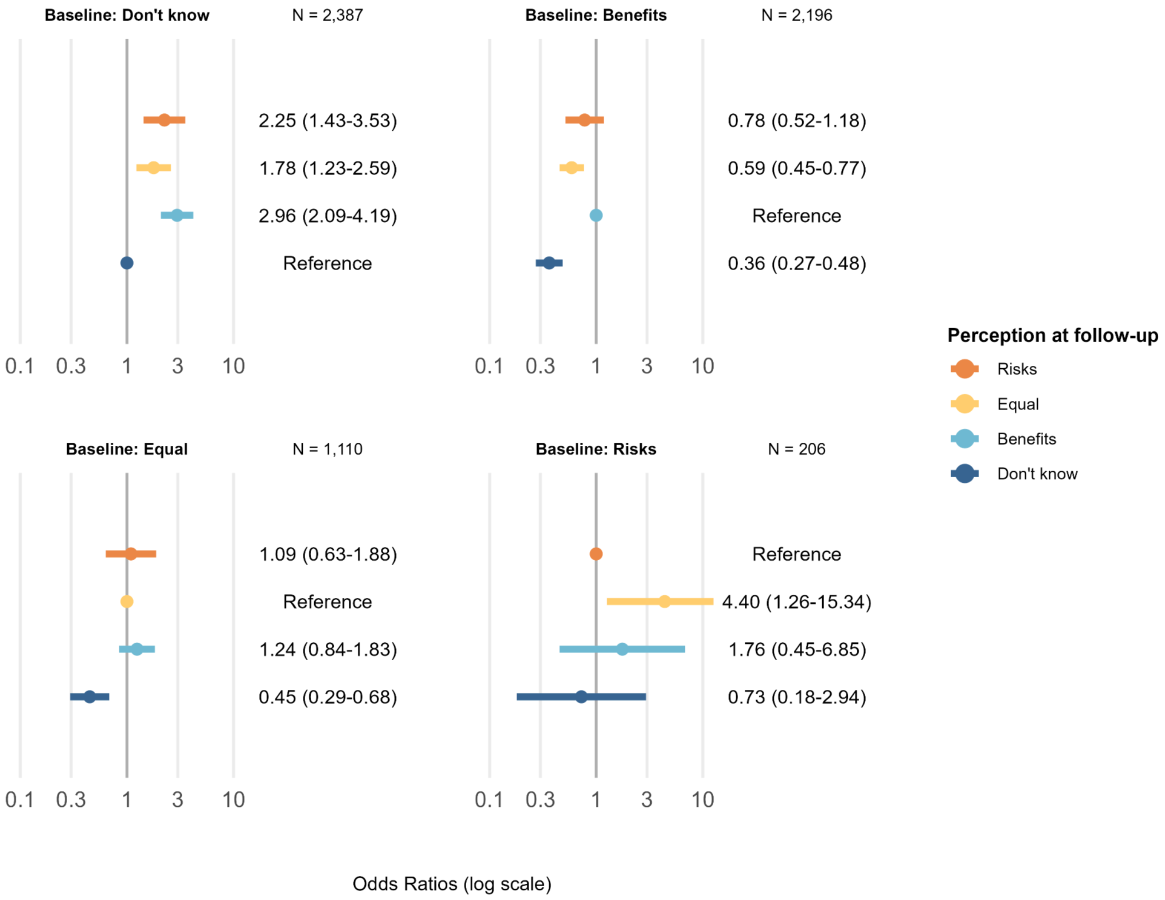

Analyses weighted to the background characteristics distribution of the Health in Central Denmark 2020 invitee population

Supplementary Table 3: Unstratified multinomial regression model additionally adjusted for baseline perception. Estimates for the association between ChatGPT exposure and perception at follow-up, with "Benefits" at follow-up as the reference outcome level.

Perception at follow-up	Estimate (95% CI)
Don't know	0.35 (0.29-0.42)
Equal	0.64 (0.54-0.76)
Risks	0.74 (0.58-0.96)

Weighted to background population characteristics distribution

Supplementary Note 1: PubMed search string for existing literature on the subject

As of 1 Dec 2024, this search query retrieves 12 results, none of which are relevant to the research question:

("artificial intelligence"[Title/Abstract] OR "machine learning"[Title/Abstract]) AND perception[Title/Abstract] AND (population[Title/Abstract] OR public[Title/Abstract] OR people[Title/Abstract]) AND ("follow-up"[Title/Abstract] OR "longitudinal"[Title/Abstract])
